# Burnout, PTSD, and Medical Error: The Medico-Legal Implications of the Mental Health Crisis Among Frontline Healthcare Professionals During COVID-19

**DOI:** 10.3390/medicina62020305

**Published:** 2026-02-02

**Authors:** Sorin Hostiuc, Florentina Gherghiceanu

**Affiliations:** 1 Department of Legal Medicine and Bioethics, Carol Davila University of Medicine and Pharmacy, 042122 Bucharest, Romania; 2 Department of Marketing and Medical Technology, Carol Davila University of Medicine and Pharmacy, 050474 Bucharest, Romania; f.gherghiceanu@umfcd.ro

**Keywords:** burnout, post-traumatic stress disorder, medical errors, healthcare workers, COVID-19 pandemic

## Abstract

*Background and Objectives*: The COVID-19 pandemic has led to an unprecedented mental health crisis among workers in the healthcare field, with average burnout rates increasing from about 32% before the pandemic to 46–52% during peak times and post-traumatic stress disorder (PTSD) affecting 24–34% of frontline staff. The primary objective of this article is to synthesize evidence on the prevalence of burnout and PTSD among healthcare workers before and during the COVID-19 pandemic. The secondary objectives are: (a) to examine the mechanisms and empirical evidence linking clinician mental health to medical errors and patient safety outcomes and (b) to analyze the medico-legal implications of this relationship, including malpractice liability, institutional responsibility, and opportunities for policy reform. *Materials and Methods*: We conducted a narrative review searching PubMed (November 2025–January 2026) using predefined keyword combinations. Inclusion criteria comprised original research, systematic reviews, and meta-analyses examining mental health outcomes or patient safety among clinical staff. Data were synthesized narratively across five thematic domains. *Results*: Burnout prevalence increased from approximately 32% pre-pandemic to 46–52% during peak periods, with emotional exhaustion reaching 67.5% in some settings. PTSD rates rose to 24–34% among frontline staff, exceeding pre-pandemic levels of 15–20%, with ICU staff particularly affected (27–40%). Substantial overlap exists between conditions (86–98% comorbidity). Physician burnout is associated with 2.72 times higher odds of self-reported errors (95% CI: 2.19–3.37), with each point increase in emotional exhaustion raising the error risk by 5–11%. Mechanisms include cognitive impairment (reduced executive function, g = −0.39; impaired working memory, g = −0.36) and sleep disturbance. Malpractice litigation compounds psychological harm, increasing depression and suicidal ideation. *Conclusions*: This review, synthesizing data from over 500,000 healthcare workers, demonstrates bidirectional relationships among burnout, PTSD, and medical errors with significant medico-legal ramifications. Addressing this crisis requires systemic interventions including workload management, psychological support, blame-free reporting cultures, and policy reforms balancing accountability with recognition of system-level contributors to error.

## 1. Introduction

The mental health issues faced by healthcare professionals are now recognized as a major public health crisis, greatly affecting their well-being and patient safety [1,2]. Studies and meta-analyses conducted before and during the COVID-19 pandemic have documented high levels of burnout, anxiety, depression, insomnia, acute stress, and post-traumatic stress disorder within the medical sector. In many settings, burnout impacts approximately one-third to one-half of the staff. The pandemic has further augmented this crisis by exposing and worsening organizational flaws in the medical systems. It has served both as a catalyst and a magnifying lens, highlighting the fragile mental health of healthcare providers operating in increasingly strained and unsustainable environments [3].

It is crucial to distinguish between burnout and post-traumatic stress disorder (PTSD), even though these conditions can co-occur and have overlapping symptoms. Burnout is considered an occupational phenomenon [4], marked by emotional exhaustion, depersonalization or cynicism, and diminished professional effectiveness [5]. It typically develops gradually due to persistent workplace stress and systemic organizational issues. In contrast, PTSD arises from exposure to actual or perceived threats of death, injury, or violence, and is characterized by intrusive memories, avoidance behaviors, negative alterations in thoughts and mood, and heightened arousal [6]. Healthcare workers may develop PTSD after witnessing patient deaths, experiencing workplace violence, or participating in mass casualty events. Throughout this review, we treat these constructs as partially overlapping phenomena, which can share risk factors and may co-occur, rather than as interchangeable terms or stages of a single continuum, this being the main reason they were treated in a single manuscript. Burnout is an occupational phenomenon operationalized primarily through the Maslach Burnout Inventory (MBI) dimensions, whereas PTSD is a clinical diagnosis with specific DSM-5/ICD-11 criteria. Secondary traumatic stress and moral injury are conceptualized as distinct but related stressors that may contribute to both burnout and PTSD symptomatology.

Both conditions have been linked to the “second victim” phenomenon. Second victims are healthcare workers who experience trauma after unexpected adverse events or medical errors, feeling personally responsible for clinical results and doubting their professional abilities [7,8,9]. Focused initially on physician errors, the concept now includes the emotional and emotional cost that any member of the healthcare team experiences after traumatic or unexpected patient care events. This impact can involve intense guilt, shame, anxiety, depression, sleep disturbances, and feelings of loss of confidence [10,11]. Systematic reviews and meta-analyses reveal that second victims commonly experience high levels of troubling memories, self-directed anger, remorse, and anxiety. These symptoms often overlap with those of post-traumatic stress disorder (PTSD) and can persist for months or even longer [10,11,12]. The second victim experience is a key moment in which burnout, PTSD, moral distress/injury, and medical errors converge. When these experiences remain unresolved, they are linked to burnout, thoughts of leaving the profession, decreased performance, and impair the safety of the patient, resulting in a cycle of additional errors and negative outcomes distress [11,12].

The prevalence of burnout and post-traumatic stress disorder (PTSD) among healthcare professionals is widely described as reaching crisis levels, with significant variation across specialties, roles, and regions [13,14,15,16]. Extensive systematic reviews of physicians and other healthcare workers report overall burnout estimates that often approach or exceed 40–50%, especially during the COVID-19 pandemic [14,16,17,18]. For example, a systematic review by Rotenstein et al., on practicing physicians, found overall burnout prevalence ranging from 0% to 80.5%, mainly due to differences in definitions and measurement tools [19]. Another review of physicians during COVID-19 reported a pooled burnout prevalence of 54.6% [17], while a meta-analysis of mixed healthcare workers during the pandemic estimated an overall prevalence of 52% [17].

PTSD, though less uniformly assessed than burnout, affects a substantial number of healthcare workers. Integrative reviews of nurses show widely varying PTSD prevalence, typically from single digits to about one-third of staff, largely due to differences in measurement methods and thresholds [20,21]. Research on ICU staff during the COVID-19 pandemic revealed that about 40–50% exhibit significant PTSD symptoms, often coupled with high burnout levels [22,23].

Burnout, PTSD, and related distress increase personnel turnover, absenteeism, and reduced productivity, all of which are costly for healthcare organizations. Systematic review evidence shows nurse turnover imposes substantial replacement and onboarding costs and is associated with poorer workgroup processes, nurse outcomes, and patient outcomes, making it “very costly” overall [24]. Similar concerns are highlighted for physicians, where mental illness and substance use contribute to performance impairment inquiries and risk of error [25,26].

Preventable clinical harm itself causes significant downstream costs. Analyses of the U.S. medical liability system estimate annual costs of $55.6 billion, about 2.4% of total health spending, including indemnity, administrative expenses, and defensive medicine [27]. Defensive practices, such as additional tests, referrals, and admissions driven by fear of lawsuits, are documented across various specialties and are linked to considerable increases in Medicare spending, potentially accounting for 8–20% of beneficiary costs depending on malpractice concerns [28]. Litigation-induced defensive and evasive medical practices also negatively impact access and quality, with clinicians avoiding high-risk patients or procedures [28,29,30].

Litigation and fear of lawsuits are closely tied to psychological harm and suicidal risk. Reviews show malpractice suits trigger prolonged anxiety, depression, anger, and sometimes suicide among physicians, fitting within second-victim and burnout pathways [29,31]. Extensive studies in physicians and surgeons demonstrate robust associations between burnout, depression, PTSD, medical errors, and suicidal ideation [32,33,34]. Perceived medical errors and malpractice fears independently predict suicidal ideation, with depressive symptoms mediating much of this effect [34,35].

The intersection of clinician mental health and medical errors has significant medico-legal implications that extend beyond individual malpractice claims. When healthcare workers operate under conditions of chronic exhaustion and psychological distress, questions arise regarding the attribution of liability: to what extent should individual clinicians bear responsibility for errors that occur within systems that failed to protect their mental health? Traditional tort frameworks emphasize individual negligence, yet emerging evidence suggests that institutional factors, such as including inadequate staffing, excessive workloads, and insufficient psychological support, sometimes causing real, objectifiable medical disoders, are primary drivers of both clinician distress and error risk. In addition, mlpractice litigation has been shown to augment psychological harm among involved clinicians, creating a vicious cycle that current legal structures may inadvertently perpetuate. These considerations have prompted calls for policy reforms including crisis standards of care, safe harbor provisions, and just culture frameworks that distinguish system-induced errors from individual negligence. 

The primary objective of this article is to synthesize evidence on the prevalence of burnout and PTSD among healthcare workers before and during the COVID-19 pandemic. The secondary objectives are: (a) to examine the mechanisms and empirical evidence linking clinician mental health to medical errors and patient safety outcomes and (b) to analyze the medico-legal implications of this relationship, including malpractice liability, institutional responsibility, and opportunities for policy reform.

## 2. Materials and Methods

To this purpose, we have performed a narrative review using primarily data from Pubmed, based on the following sets of keywords (see Table 1). The primary search was conducted between 15 November 2025 and 15 December 2025, with an update after the initial peer review, up to 24 January 2026. To identify additional relevant studies, we further scrutinized the reference lists of the retrieved articles and used Consensus.app 2.0 (Consensus NLP, Inc. (Boston, MA, USA)) and Google Scholar Labs (Experimental beta, Google LLC (Mountain View, CA, USA)) to identify similar studies. These AI-assisted discovery tools were used as supplementary methods after the primary PubMed search was completed; they helped identify additional relevant studies that were then independently screened against our predefined inclusion criteria. The AI-assisted discovery served to expand, not replace, systematic screening. When multiple systematic reviews or meta-analyses addressed the same association, we prioritized the most recent and comprehensive review while noting instances of concordant or discordant findings. When contradictory findings emerged, we presented both perspectives and discussed potential sources of heterogeneity. A narrative synthesis was chosen over systematic review methodology because our aim was to integrate evidence across heterogeneous research domains (psychological outcomes, patient safety metrics, medico-legal frameworks) that employ fundamentally different methodologies and outcome measures, making formal meta-analytic pooling inappropriate for the overarching synthesis. We limited our initial search to 2015–2025, with relevant, older articles being gathered using the secondary methods listed above.

We used the following general inclusion criteria: original research articles (cross-sectional, cohort, longitudinal studies), systematic reviews, and meta-analyses; studies examining burnout, PTSD, or medical errors among healthcare workers; studies published in English; studies providing quantitative prevalence data or examining relationships between mental health and patient safety outcomes. The exclusion criteria included: conference abstracts, editorials, letters, and commentaries without original data; studies focusing exclusively on non-clinical healthcare staff (administrative personnel only); case reports involving fewer than 10 participants; studies not specifically addressing healthcare worker populations; non-peer-reviewed publications (preprints without subsequent publication). Both authors independently screened titles and abstracts for relevance. Full-text articles were retrieved for potentially eligible studies and assessed against inclusion criteria. Disagreements were resolved through discussion. Given the narrative nature of this review, formal quality assessment using standardized tools (e.g., Newcastle-Ottawa Scale, GRADE) was not systematically applied. However, priority was given to systematic reviews, meta-analyses, and large-scale multicenter studies. Studies with sample sizes below 100 were included only when addressing unique populations or regions with limited data. The thematic analysis was organized around the following predefined domains: (1) prevalence and characteristics of burnout before and during the pandemic, (2) prevalence and characteristics of PTSD before and during the pandemic, (3) mechanisms linking mental health to medical errors, (4) empirical evidence of the mental health–error relationship, and (5) legal and systemic implications. Data were synthesized narratively, with quantitative findings from meta-analyses presented where available.

## 3. Burnout in Healthcare Professionals

Burnout, as understood among healthcare professionals, is defined as a work-related syndrome caused by chronic occupational stress that has not been properly managed. The most widely accepted operational definition comes from the Maslach Burnout Inventory (MBI), which assesses three distinct but related dimensions: emotional exhaustion, depersonalization, and reduced personal accomplishment [5]. Emotional exhaustion involves feeling emotionally drained and depleted by one’s work—where emotional resources are exhausted and can no longer provide the necessary attention and care to patients. Healthcare workers experiencing emotional exhaustion report feeling drained, used up, frustrated, and overwhelmed by the sense that they are working too hard [36]. The second dimension, depersonalization (or cynicism in non-healthcare contexts), leads to negative or inappropriate attitudes toward patients, detached concern, irritability, loss of idealism, or withdrawal from meaningful engagement with those in need of assistance [37]. It functions as a defensive coping mechanism in which healthcare professionals distance themselves emotionally from their patients to manage overwhelming demands; however, this distance actually undermines the compassionate care that defines healthcare professionals [38]. The third dimension, reduced personal accomplishment (or professional efficacy), is characterized by declining feelings of competence and success in one’s work. Healthcare professionals experiencing this dimension evaluate themselves negatively, feeling unhappy, dissatisfied with their achievements, which contributes to decreased productivity, guilt, low morale, and an inability to cope [5].

The inclusion of burnout in the 11th revision of the ICD marks a significant milestone in formally recognizing burnout as an occupational phenomenon [4]. ICD-11 defines burnout as an occupational disorder resulting specifically from chronic workplace stress that has not been successfully managed, characterized by feelings of energy depletion or exhaustion, increased mental distance from one’s job or feelings of negativism or cynicism related to one’s job, and reduced professional efficacy [39].

Burnout in healthcare is now recognized as a threat to the “quadruple aim” because it undermines clinician wellbeing, degrades patient experience and quality, and increases costs at the system level [17,40]. Systematic reviews describe burnout not as a static end state but as a dynamic loss spiral, where emotional exhaustion, depersonalization, and diminished accomplishment progressively damage personal health, professional functioning, and organizational stability [17,41]. In particular, emotional exhaustion appears to be the burnout dimension most strongly associated with adverse outcomes, including psychological distress and impaired performance [17,40]. 

At the personal level, burnout is strongly linked to various health risks. Studies consistently show that physician burnout correlates with depression, anxiety, and higher suicidal thoughts, with suicide rates among physicians exceeding those in the general population—especially in frontline specialties and among women [41,42]. Large surveys in the U.S. reveal that 12.9% of male physicians and 21.4% of female physicians meet criteria for alcohol abuse or dependence, and problematic alcohol use is significantly tied to burnout, depression, suicidal thoughts, and recent medical errors [42,43]. Mixed-method and narrative reviews indicate that some clinicians use alcohol and other substances as maladaptive coping mechanisms to reduce distress or self-treat pain and anxiety, even when a direct, consistent causal link to burnout is hard to prove [41,44]. Across healthcare disciplines, higher burnout and secondary traumatic stress are linked to increased non-medical use of prescription drugs, cannabis, and other illicit substances, with lower-wage healthcare workers being especially vulnerable [45]. Clinicians experiencing burnout often report irritability, fatigue, and emotional withdrawal, which can strain personal and family relationships and lead to social isolation [17,42,46].

Professional functioning and the quality of patient care are also negatively affected. Meta-analyses and systematic reviews indicate that higher burnout levels among physicians and other healthcare workers are associated with increased patient safety incidents, diminished professionalism, and lower patient satisfaction. Estimates show that safety events and unprofessional behaviors roughly double in physicians experiencing high burnout compared to their less-burned-out counterparts [46,47]. Clinicians facing burnout often report a lower perceived quality of care, more self-reported medical errors, and increased emotional detachment from patients. They frequently describe viewing patients as objects rather than individuals, along with a loss of empathy and meaning in their work [17,40,46]. Among trainees, higher burnout is associated with reduced empathy, altruism, and honesty, as well as problematic patient care practices, suggesting early erosion of core professional values [48]. 

At the organizational level, burnout leads to absenteeism, lower productivity, increased intentions to leave, and actual turnover, all causing significant economic and operational challenges for healthcare systems [40,42,46]. Meta-analyses show that burned-out physicians are several times more likely to have low job satisfaction, regret their career choices, consider leaving, and reduce clinical efforts, threatening workforce sustainability [47]. High burnout rates in institutions are linked to higher malpractice risks, more medical errors, and poorer team functioning and organizational commitment, creating a vicious cycle where worsening work conditions lead to more burnout [42,44,49]. Overall, these personal, professional, patient-care, and organizational consequences demonstrate that clinician burnout is not just an individual resilience issue but a systemic problem that endangers both individual well-being and the effective functioning of healthcare systems [42,47,50].

### Burnout Characteristics Before and During the Pandemic

Prior to the COVID-19 pandemic, burnout among healthcare workers was already recognised as a significant occupational health concern, though prevalence estimates varied considerably depending on measurement instruments, threshold definitions, and populations studied. Rotenstein et al. (2018) conducted the most comprehensive systematic review of physician burnout, encompassing 182 studies involving 109,628 physicians across 45 countries, and reported prevalence rates ranging from 0% to 80.5%, with this remarkable heterogeneity attributed primarily to inconsistent definitions and measurement approaches [19]. Despite this variability, converging evidence suggested that approximately 30–50% of physicians experienced at least one dimension of burnout as measured by the Maslach Burnout Inventory [51,52]. Certain specialties demonstrated consistently elevated baseline rates: emergency medicine physicians exhibited burnout prevalence of 45.8–60%, the highest among medical specialties, driven by high-acuity patient loads, shift work, and frequent exposure to traumatic events [51,53]. Intensive care unit professionals reported pre-pandemic burnout rates of 25–41%, with emotional exhaustion affecting approximately 40% of ICU staff [14,54]. Primary care physicians demonstrated prevalence rates of 40–54%, attributed to increasing administrative burdens, electronic health record demands, and time pressures limiting patient interactions [19,55]. Among nurses, pre-pandemic data indicated that 30–43% experienced high emotional exhaustion, with ICU nurses reporting rates of 33–40% [54,56].

The COVID-19 pandemic has significantly increased burnout among medical personnel, reaching values above the pre-pandemic levels and introducing new, intense stressors, which have modified its characteristics. Before COVID-19, burnout was already a major concern, with estimates generally between 30% and 50%, varying by country, specialty, and measurement methods. For example, a large cross-sectional study in Iran before the pandemic found that nearly half of healthcare workers experienced moderate to high burnout, with emotional exhaustion and depersonalization particularly prevalent among nurses in high-stress roles [51]. The pandemic worsened these figures considerably. A systematic review of 50 studies with 30,000 healthcare workers reported burnout rates up to 75% in some areas, particularly in the Middle East and among frontline staff [52]. In Singapore, a survey conducted three months after the outbreak was declared found that over 75% of healthcare workers experienced exhaustion-related burnout, and nearly 80% met criteria for disengagement, with redeployment and longer shifts as key risk factors [53]. Comparing studies and repeated surveys provide direct evidence of increased burnout from pre-pandemic to pandemic times. In the U.S., burnout rose from 32% in 2018 to 46% in 2022, with emotional exhaustion increasing from 31.8% in 2019 to 40.4% in 2021–2022 [57]. In Catalonia, Spain, repeated surveys of general practitioners indicated that emotional exhaustion peaked at 67.5% during the first pandemic wave, then declined slightly but remained above 56% in subsequent years, indicating high, persistent burnout despite easing COVID-19 pressures [58]. Similarly, an Italian study during the first wave found 38.3% of healthcare workers with high emotional exhaustion, with higher rates in ICU staff and residents [59]. A 2022 survey in Poland found 36.5% of nurses and 27.7% of non-medical staff met burnout criteria, with stress, traumatic work experiences, and increased workload as key predictors [60]. See Table 2 for a summary of key meta-analyses on COVID-19-related burnout metrics.

The pandemic not only increased burnout but also changed its main characteristics, by emphasizing moral injury and ethical distress as core issues. Medical personnel often faced impossible choices, such as rationing ventilators or reusing personal protective equipment (PPE), and were not infrequently working under conditions that conflicted with their professional standards and values [71,72]. These experiences resulted in “moral residue,” the lingering psychological effects of repeated moral violations and unresolved ethical dilemmas [73]. A qualitative study involving NHS mental health staff in England identified moral injury as a key aspect of their pandemic experience, with feelings of guilt, helplessness, and distress over their inability to deliver adequate care, worsened by poor communication and organizational support [71]. Quantitative data also highlights a strong link between moral distress and burnout, with each fueling the other and both predicting intentions to leave the profession [74].

During the pandemic, differences in burnout across specialties and demographics became more evident. Emergency medicine, intensive care, and primary care providers, which were already at higher risks, were especially affected as they operated on the COVID-19 frontline, facing increased viral exposure, high mortality, and moral distress [68,75]. Nurses and women healthcare workers consistently reported higher burnout, emotional exhaustion, and psychological distress, reflecting ongoing disparities and pandemic-related burdens. In Singapore, nurses and women were at higher risk of burnout, with redeployment to unfamiliar roles further increasing their risk [76]. 

Younger healthcare workers and those with less experience were also more vulnerable, with burnout linked to intentions to leave the profession and to poor work performance [77,78]. The psychological aftermath of pandemic burnout is significant and long-lasting. Studies from Australia and elsewhere show that, besides burnout, healthcare workers face high levels of anxiety (60%), depression (57%), and post-traumatic stress symptoms. Over three-quarters report negative effects on their relationships with family, friends, and colleagues [79]. In South Korea, over 77% of healthcare workers met burnout criteria, with chronic fatigue, physical symptoms, and post-traumatic stress as key correlates [80]. The cycle of moral distress and burnout fostered feelings of entrapment, with workers feeling both duty-bound and unable to deliver care at professional standards, leading to guilt, shame, and emotional exhaustion [71,73]. Although some data indicate slight improvements in burnout as COVID-19 cases and their perceived severity decreased, systemic issues such as chronic staffing shortages, underinvestment in workforce well-being, administrative overload, and a focus on efficiency over quality have mainly remained unresolved [78,81].

## 4. Posttraumatic Stress Disorder in Healthcare Workers

Post-traumatic stress disorder (PTSD) is classified as a trauma- and stressor-related condition in current diagnostic manuals. It develops following exposure to severe stressors like actual or threatened death, serious injury, or sexual violence [82,83,84]. According to DSM-5 and ICD-11, the diagnosis is focused on key symptom groups, including intrusive re-experiencing of the trauma, avoidance of reminders, negative changes in thoughts and mood, and significant shifts in arousal and reactivity. These symptoms must last for at least one month, should lead to significant distress or functional impairment [82,83,85], and are prone to lead to various physical, psychological, social, and occupational problems, such as strained relationships, decreased productivity, increased healthcare use, and a heightened risk of suicidal thoughts and actions [86,87].

For healthcare professionals and other first responders, experiencing traumatic events that qualify for PTSD can occur through various recognized pathways aligned with DSM-5 criterion A. PTSD develops following exposure to situations that threaten life, cause serious injury, or involve sexual violence. These can happen through direct involvement, witnessing the event firsthand, learning about a close family member or friend’s violent or accidental death, or through repeated or extreme indirect exposure to traumatic details as part of professional duties [83,88]. This last pathway often includes roles such as first responders handling human remains, police officers frequently exposed to child-abuse materials, and healthcare workers regularly dealing with severe injuries, resuscitations, and deaths [88,89,90].

Emergency and critical care professionals, such as paramedics and first responders, are particularly vulnerable to repeated or intense exposure to traumatic details. Still, other specialties have higher risks as well, including psychiatry, gynecology, surgery, pediatrics, etc. [91,92,93,94,95,96,97,98]. Their daily duties often include managing life-threatening emergencies, witnessing serious injuries and fatalities, and routinely facing suffering and violence. Systematic reviews show that professionals such as healthcare workers, firefighters, police officers, and military personnel have notably higher PTSD rates than the general public. This heightened risk stems from their regular exposure to potentially traumatic incidents and the nature of their work [89,90,99,100].

PTSD should be distinctly distinguished from acute stress disorder (ASD), vicarious trauma, and secondary traumatic stress (STS).

Acute stress disorder (ASD) is defined in DSM-5 as an early trauma- and stressor-related disorder, sharing many symptoms with PTSD, such as intrusive memories, avoidance, negative mood, dissociation, and hyperarousal [101,102]. The main difference lies in timing: ASD is diagnosed when symptoms occur between three days and one month after a traumatic event; if the symptoms persist beyond a month, PTSD is the more appropriate diagnosis [101,103]. For a time, ASD was used to identify individuals at higher risk of developing PTSD later, but longitudinal studies have proven that, although ASD increases the risk, many people who develop PTSD do not exhibit ASD symptoms within the first month after trauma [101,102,104].

Vicarious trauma and STS are related but distinct concepts describing how professionals are impacted by indirectly experiencing others’ trauma. Vicarious trauma, based on the constructivist self-development theory, involves slow, lasting changes in core beliefs about safety, trust, control, self-esteem, and intimacy. These changes typically develop gradually through repeated exposure to clients’ traumatic stories [105,106,107]. Usually, these symptoms shift over time, reflecting rather a continuous empathic engagement with trauma narratives rather than a single incident, and can lead to distrust, hopelessness, or altered worldviews among healthcare workers, psychotherapists, and social workers [105,107,108]. STS, unlike vicarious trauma, centers on the symptoms that arise from indirect exposure to trauma. It is marked by PTSD-like signs such as intrusive thoughts, avoidance, negative feelings or beliefs, and hyperarousal, which come from witnessing others’ traumatic events rather than experiencing trauma directly [106,107,109,110]. STS can develop rapidly and may result from brief or intense exposures, like working with a few highly distressing cases [111]. Studies on health professionals show that STS mimics the full PTSD symptom cluster, including intrusive images, sleep disturbances, irritability, fatigue, and concentration problems, which can cause substantial occupational and personal challenges [107,109,112].

Conceptually, PTSD and ASD require exposure that meets DSM Criterion A, which may include indirect professional exposure to traumatic details such as repeated encounters with injury or death in emergency medicine. STS and vicarious trauma are specifically characterized by their origins in indirect, work-related exposure within helping relationships [101,103,107]. Recent network analyses indicate that, although STS and vicarious trauma overlap, they are distinct: STS features acute PTSD-like symptoms, while vicarious trauma involves broader changes in cognition and beliefs [106,113]. For healthcare workers, indirect exposure can occur through listening to detailed victim accounts, examining disturbing clinical images or case files, or repeated contact with severe suffering, violence, and death, all potentially leading to STS, vicarious trauma, burnout, and sometimes full PTSD.

Healthcare professionals face various sources of trauma, including individual critical incidents and ongoing, cumulative stress. Frequent exposure to death, especially in high-mortality settings like intensive care and palliative care, is now viewed as repeated exposure to potentially traumatic vicarious events rather than just routine professional experiences. Longitudinal research with palliative care staff shows that continuous end-of-life work is a major chronic stressor, with about one-third experiencing ongoing distress rather than resilience. This chronic stress is the strongest predictor of mental health outcomes over time [114]. Qualitative and review findings indicate that patient deaths, especially when perceived as prolonged, futile, or “not a good death”, are key sources of moral distress and professional grief, affecting personal life, team cohesion, and career decisions [115,116,117]. Systematic reviews across various specialties consistently find that repeated exposure to patient suffering and death, emotional exhaustion, or low job satisfaction are linked to secondary traumatic stress symptoms in healthcare workers [118,119].

Acute medical emergencies and critical incidents cause high stress among providers, which can negatively impact cognition, technical skills, and team coordination. Data from trauma surgery and high-stakes environments indicate that acute stress hampers decision-making, memory, and psychomotor skills, raising the likelihood of errors, mainly when clinicians face life-or-death decisions under pressure and uncertainty [120]. In cases involving serious harm, unexpected death, or visible procedural issues, clinicians may become “second victims,” experiencing intense guilt, shame, intrusive memories, and self-doubt. A systematic review of second-victim experiences shows lasting negative psychological effects, including symptoms similar to PTSD, but support through debriefing and organizational backing can lessen the long-term impact [121]. A scoping review done by Harder et al. on debriefing after patient deaths find that emotionally focused debriefings are linked to better psychological outcomes, though they are not consistently used after distressing events [116].

Moral distress and moral injury represent distinct trauma pathways in healthcare, especially concerning life-and-death choices, triage, and resource allocation. Intensive care physicians report moral distress caused by perceived treatment futility, pressure to continue non-beneficial interventions, and restrictions on clinical autonomy during end-of-life decisions. These experiences often lead to ongoing feelings of regret, anger, and a sense of failing to ensure a “good death” for patients [115].

More broadly, moral injury is characterized by an enduring distress after potentially morally injurious events (PMIEs), such as being forced by systemic constraints to violate one’s fundamental ethical principles. Studies indicate that healthcare workers face PMIEs more often than military personnel, due to issues like chronic understaffing, care rationing, and organizational policies that impede optimal care [122,123]. Large UK COVID-19 healthcare surveys show a strong connection between PMIE exposure and higher incidences of PTSD, depression, and suicidality, especially among those who were redeployed, inadequately protected, or who experienced the loss of colleagues [124]. Also, reviews regarding the physicians’ mental health during the pandemic have strongly emphasised the significant impact of issues such as moral injury, burnout, grief, and uncertainty stemming from triage, rationing, and witnessing preventable deaths in overwhelmed systems [125].

Workplace violence from patients, relatives, and colleagues is a common and often normalized source of trauma in healthcare. A large meta-analysis involving 331,544 healthcare workers found that about 62% experienced some form of violence in the past year, with 24% facing physical assaults and nearly 58% verbal abuse. These rates were highest in emergency, psychiatric, and intensive care settings, particularly among nurses and physicians [126]. More recent research supports not only the high prevalence but also the serious psychological effects, such as PTSD symptoms, hypervigilance, avoidance, and increased use of psychotropic medications and therapy among victims [127,128]. A systematic review focusing on female healthcare workers estimates a 45% pooled prevalence of workplace violence, including verbal abuse, threats, physical assaults, sexual harassment, and bullying, all linked to negative mental and physical health outcomes [129]. Qualitative studies in psychiatric hospitals reveal that staff are traumatized not only by direct assaults but also by vicarious exposure to patients’ traumatic histories and suffering. The lack of organizational support and the message that violence is “part of the job” intensify psychological harm, contributing to PTSD and emotional exhaustion [130]. Importantly, cross-sectional data suggest that even indirect exposure, including media coverage of violent attacks on clinicians, may increase PTSD, depression, and anxiety symptoms in healthcare workers by raising risk perception [131].

Beyond isolated incidents, healthcare workers also face cumulative secondary and vicarious trauma from ongoing interactions with trauma survivors. Evidence from cross-sectional studies and reviews shows that paramedics, nurses, emergency physicians, and other staff working with trauma victims frequently experience secondary traumatic stress levels that are comparable to or even higher than those of directly affected trauma survivors. Nearly half of some samples meet criteria for probable secondary PTSD [114,118,119]. Factors contributing to this include high trauma caseloads, emotional exhaustion, lack of sufficient social and organizational support, and maladaptive cognitive strategies like persistent regret and rumination. Conversely, self-care, peer support, psychological flexibility, and debriefing are linked to more resilient outcomes [114,118,119].

### 4.1. The Overlap Between PTSD and Burnout

PTSD and burnout often occur together among healthcare workers. Cross-sectional studies on nurses reveal a very high overlap: at a US university hospital, 86% of nurses diagnosed with PTSD also experienced burnout, and 98% of those with PTSD met burnout criteria, suggesting PTSD rarely exists without burnout in this group [132]. Similar trends are seen in different settings. During COVID-19, only 3% of intensive care staff had PTSD alone, while 16% experienced both PTSD and burnout, with those individuals reporting the highest work-related stress [23]. Emergency and ICU staff also show that those with PTSD tend to have higher burnout scores and more overall impairment, highlighting the added challenge of the combined conditions [22,89].

Many risk factors associated with burnout are also present in healthcare workers suffering from PTSD. Workplace violence more than doubles nurses’ chances of developing both PTSD and burnout, drawing attention to a common pathway through traumatic exposure and ongoing threat [42]. Psychiatric and emergency healthcare workers exposed to critical incidents, distressing patient behaviors, high workloads, and poor work–life balance show similar increases in burnout and PTSD symptoms, with burnout partly mediating the link between critical events and PTSD [133]. Large COVID-19 cohorts reveal that heavy workloads, insufficient resources, fear of infection, female gender, younger age, and lack of organizational and social support are associated with an increased risk for both burnout and post-traumatic symptoms [134,135]. Personal traits and pre-existing vulnerabilities like prior anxiety, depression, or mood-spectrum issues further increase the risk for both conditions and are connected to greater functional impairment when they occur simultaneously.

There seems to be a bidirectional and potentially causal link between PTSD and burnout. Long-term studies of healthcare workers started from pre-COVID times through the pandemic have shown that higher emotional exhaustion before the pandemic and worsening exhaustion and depersonalisation strongly predict later PTSD symptoms. This suggests that burnout-related depletion of psychological resources makes individuals more vulnerable to trauma-related disorders during major crises [136]. Follow-up research on frontline COVID-19 workers indicates that ongoing burnout contributes to acute stress, depersonalisation/derealisation, and indirectly to PTSD symptoms, with high resilience offering a protective effect [137].

PTSD can also promote burnout progression as was shown, for examples, by studies about Iranian and Chinese nurses during COVID-19, which have found that PTSD severity significantly predicted higher burnout levels, this being the only independent predictor in multivariate models, and therefore implying that PTSD-related symptoms can diminish occupational engagement and energy [138,139]. In psychiatric hospitals, burnout not only correlates directly with PTSD symptoms but also alters the impact of critical workplace events on PTSD, suggesting the presence of a complex feedback loop between trauma exposure, chronic stress, and exhaustion [133].

When PTSD and burnout occur together, their combined effect on clinicians and healthcare systems exceeds the sum of their individual impacts. Nurses experiencing both report notably poorer perceptions of teamwork, decreased trust in physicians, and greater work–life interference than those with burnout alone [132]. Emergency and ICU staff with both conditions exhibit greater overall functional impairment, lower job satisfaction, and a stronger desire to leave, raising concerns about staffing retention [22,140]. During COVID, studies consistently link high burnout and trauma symptoms with increased anxiety, depression, insomnia, and distress, which may impair clinical performance and raise the risks of substance use and suicidality [135,141]. Social support and psychological resilience can partly buffer the connection between burnout and PTSD, emphasizing the importance of organizational strategies such as improving perceived support, ensuring adequate staffing and protection, and fostering positive team environments, alongside individual treatments targeting trauma and burnout [131,134,141].

### 4.2. PTSD in Healthcare Workers Before and During the COVID-19 Pandemic

Before the COVID-19 pandemic, post-traumatic stress disorder among healthcare workers was recognised but comparatively understudied relative to burnout. General population lifetime PTSD prevalence in high-income countries was in general reported as below or around 10%. For example, Kilpatrick et al. reported a 9.4% composite event lifetime PTSD according to the DSM-5 criteria [142]. Pre-pandemic studies consistently demonstrated elevated PTSD rates among healthcare workers compared to the general population, with estimates ranging from 10% to 20% depending on clinical setting and exposure to traumatic events [132,143]. Nurses working in general hospital settings exhibited PTSD prevalence of approximately 14–18%, while intensive care unit nurses demonstrated higher rates of 18–24%, reflecting their routine exposure to patient suffering, death, and ethically challenging end-of-life decisions [132,144]. Among ICU professionals broadly, a narrative review identified pre-pandemic PTSD prevalence ranging from 3.3% to 24%, with this variability reflecting differences in measurement instruments, diagnostic thresholds, and healthcare system contexts [145]. Previous coronavirus outbreaks provided important precedents: healthcare workers involved in the 2003 SARS outbreak demonstrated acute PTSD rates of 10–20%, with long-term follow-up studies revealing persistent symptoms in 5–18% of affected staff up to four years post-outbreak [146,147]. Similar results were found in healthcare workers during the 2015 MERS outbreak in South Korea, which exhibited PTSD prevalence of 15–20% [148].

The COVID-19 pandemic dramatically changed how PTSD affects healthcare workers, with major reviews showing a clear increase compared to pre-pandemic times and earlier outbreaks (see Table 3). A recent systematic review and meta-analysis of 119 studies involving 117,143 healthcare workers over more than two years estimated a pooled PTSD symptom prevalence of 34% (95% CI 30–39%) and severe PTSD at 14% (95% CI 11–17%), significantly higher than pre-COVID estimates in similar populations and early 2020 meta-analyses, which reported rates around 20–27% [149]. An umbrella review of meta-analyses on healthcare workers reported a slightly more cautious pooled PTSD prevalence of 13.5% (95% CI 9.1–18.0%), but still confirmed higher rates during COVID-19 compared to earlier data, where PTSD prevalence ranged from 21.5% to 38% depending on setting and measurement [150]. Meta-analyses covering pandemics since 2000 also indicate that healthcare workers exhibit the highest post-pandemic PTSD rates among all groups—26.9% (95% CI 20.3–33.6%), rising to 31% in frontline staff—compared to 19.3% in the general public, highlighting the disproportionate impact on this group [151].

Nurses stood out as a subgroup particularly affected by trauma. A comprehensive review and meta-analysis of 55 studies from 26 countries, involving over 40,000 nurses, estimated a pooled PTSD prevalence of 29.1% (95% CI 23.5–35.5%) during COVID-19, nearly double the 15% prevalence reported for the general population during the same period [155]. Another meta-analysis of 28 studies with 10,074 healthcare workers estimated PTSD rates of 31% (95% CI 21–40%) in physicians and 38% (95% CI 30–45%) in nurses, highlighting nurses’ increased vulnerability [156]. National-level data show similar trends: in Chinese healthcare workers, pooled PTSD prevalence during COVID-19 was 29.2% (95% CI 20.7–33.7%) across 21 studies with 11,841 participants [157]. Regional reviews reveal wide variability driven by context: in sub-Saharan Africa, healthcare workers’ pandemic-period PTSD ranged from 11.7% to 78.3% across studies, with most reporting much higher rates than pre-COVID levels [158]. Hospital-based studies conducted during early peaks support these findings: for example, a UK survey of 2638 hospital staff in June–July 2020 found 24.5% exhibited clinically significant PTSD symptoms, with 34.3% experiencing anxiety and 31.2% depression [159]. A Canadian study three months into the first wave reported probable PTSD in 24.3% of respondents and over half experiencing frequent burnout [134]. More recent cross-sectional research in critical care shows that even later in the pandemic, about 27% of intensive care providers still met criteria for probable PTSD, with very high rates of depression (67%), anxiety (63%), and burnout (over 50% across domains) [160].

Meta-analyses and large cohort studies show that the pandemic heightened both traditional trauma-related risk factors and new, COVID-specific stressors. For healthcare workers overall, combined analyses identify female gender, nursing roles, frontline positions, long working hours, assignments to isolation or COVID wards, high exposure to infected or critically ill patients, inadequate personal protective equipment (PPE), fears of infection or transmitting the virus to family, and pre-existing mental health conditions as consistent PTSD predictors [150,151,156]. Nurse-focused meta-analyses detail these patterns: prior mental health issues, female gender, high contact with COVID-19 patients, insufficient protective measures, and heavy workloads all significantly increased PTSD risk [155]. A Polish survey of 852 healthcare workers reported high PTSD symptoms (mean PCL-C 37.9), with women and nurses showing notably higher avoidance and intrusion scores. Regression analysis revealed that fear of personal health was the strongest individual PTSD predictor (β = 0.15, *p* < 0.001), accounting for 11% of outcome variance [161]. Early-wave ICU and hospital cohort studies highlight the importance of modifiable workplace factors: adequate PPE availability, perceived organizational support, and lower exposure to moral dilemmas all independently correlated with significantly reduced PTSD, anxiety, and depression [134,159].

It is interesting to mention that the introduction of COVID-19 vaccines led to a significant decrease in pooled PTSD prevalence, while the emergence of new variants caused smaller, temporary increases [149]. Systematic reviews of coronavirus outbreaks (SARS, MERS, COVID-19) consistently identify higher social and organizational support, proper training, clear communication, increased professional recognition, and greater resilience as factors that buffer against PTSD or reduce postoperative stress symptoms [151,162,163]. In a machine learning study involving 437 Brazilian healthcare workers, stress related to social isolation and low professional recognition during COVID-19 were the strongest predictors of PTSD and depression severity, while higher perceived recognition had a strong protective effect [163].

Multiple reviews have shown that COVID-19 was associated with trauma features distinct from typical workplace exposures. Instead of isolated, short-term critical incidents, healthcare workers endured extended, unpredictable exposure to mass death, high mortality with limited effective treatments, and a constant threat of contagion to themselves and loved ones [89,151]. Some meta-analyses, which have also evaluated earlier outbreaks, have shown that PTSD rates during COVID-19 were higher compared to those after SARS, H1N1, or Ebola outbreaks, due to factors like the widespread incidence of SARS-CoV-2, PPE shortages, intense media coverage, ongoing uncertainty, and moral stress related to triage and resource scarcity [149,151]. Systematic reviews of PTSD symptoms in COVID-19 healthcare workers show frequent re-experiencing of patient deaths and moral injury from enforcing isolation, participating in decisions about withdrawing life support, and witnessing patients die without family present [164,165]. Coleman et al. have shown that around 24% of medical personnel had PTSD-related IES-R scores (≥24) by 2023, with those in a chronic distress trajectory nearly seven times more likely to develop PTSD than resilient colleagues, highlighting lasting effects beyond initial peaks [166]. Consistent with long-term data from SARS and MERS, meta-analyses suggest that PTSD in healthcare workers can persist for months or years, with frontline staff still showing around 29% prevalence within six months after outbreaks and observing notable delayed-onset PTSD in some groups [151,167].

## 5. The Link Between Mental Health and Medical Error

Medical errors encompass a range of preventable adverse events that occur during healthcare. The 1999 Institute of Medicine report, To Err Is Human, classifies these errors into the following categories: diagnostic errors (missed, delayed, or incorrect diagnoses), treatment errors (wrong procedures or improper execution), medication errors (wrong drug, dose, or route), surgical errors, and communication failures between providers or with patients [168]. A crucial distinction exists between near-misses, namely errors caught before reaching the patient, and adverse events that cause harm. Additionally, medical jurisprudence often differentiates between errors (unintentional acts or omissions) and negligence (failure to meet the standard of care), although this distinction can be blurred in legal contexts [169].

Medical errors continue to be a leading cause of preventable illness and death worldwide. In the U.S., Makary and Daniel estimated that these errors might rank as the third most common cause of death, resulting in over 250,000 deaths each year [169].

The COVID-19 pandemic increased the severity of multople pre-existing issues in health systems. Rapid, unplanned organizational changes, such as expanding roles without adequate training, accelerating licensing processes, and shifting substantial care volumes to telemedicine on incomplete digital systems, have created opportunities for errors in prescribing, monitoring, and follow-up [170,171]. These structural changes were compounded by heavy workloads, high case complexity, and widespread uncertainty about optimal treatments, all of which compromised clinicians’ cognitive function and increased the risk of procedural and judgmental errors [72,172].

Diagnostic safety was especially at risk in this situation. Prior to COVID-19, missed, delayed, and incorrect diagnoses already contributed significantly to patient harm; the pandemic introduced further complexity. Gandhi and Singh described how changing knowledge about COVID-19 symptoms, compromised safety of clinicians both physically and psychologically, staffing shortages, and rapidly changing care protocols increased the likelihood of diagnostic errors throughout the healthcare process [172]. Evidence supports these concerns. A study of adult patients hospitalized as “patients under investigation” for COVID-19 across multiple US centers from February to July 2020 found diagnostic errors in 14% of cases, often attributable to issues in clinical assessment, test selection, history-taking, and physical examination. More than one-third of these patients suffered harm or death due to diagnostic errors, highlighting their clinical importance [173].

Focusing more intently on patients with confirmed COVID-19 has revealed how concurrent conditions are often overlooked. In a cohort of 1249 hospitalized COVID-19 patients at a tertiary center, 17% had a second diagnosis at presentation; for 34% of these, the second diagnosis was delayed, averaging 1.5 days [174]. Treatment was postponed in 86%, and over one-third of interventional procedures were delayed, especially infections, which are more prone to delayed detection, whereas surgical conditions were more commonly identified quickly [174,175]. Survey data from Turkey supports these findings on a clinical level: over half of the physicians surveyed believed that suspicion of COVID-19 overshadowed other diagnoses, causing initial misdiagnoses, and nearly 60% thought that conditions with similar symptoms to COVID-19 were often missed during the pandemic [176].

Delays and disruptions in access to medical care have worsened the effects of diagnostic and treatment errors. A comprehensive set of systematic reviews and meta-analyses on emergency conditions revealed that, during the COVID-19 pandemic compared to pre-pandemic times, patients experienced longer waits from symptom onset to hospital admission for acute coronary syndromes, higher incidences of vasospasm after aneurysmal subarachnoid hemorrhage, increased perforations in acute appendicitis, more cases of diabetic ketoacidosis at type 1 diabetes onset, and higher orchiectomy rates in testicular torsion [177]. These signs indicate significant patient-related delays and compromised emergency care pathways. Similar issues appeared in chronic and cancer care: a review of 62 studies across various regions documented widespread reductions in routine cancer services, including lower screening attendance, diagnostic testing, and surgery, with up to 77.5% of facilities and 79% of supply chains disrupted [178]. Such delays lead to missed or late diagnoses, resulting in preventable illness and death.

The pandemic environment also impacted medication safety in specific ways. Al Meslamani pointed out that the combination of untested drugs, inexperienced or redeployed staff, and high stress created ideal conditions for prescribing and administering errors [170]. A cross-sectional study of nurses in Iranian teaching hospitals during COVID-19 revealed that over one-third reported at least one medication error in the past year, with wrong-patient and wrong-dose errors being the most common. These errors were significantly linked to shift work, and fear of reporting was identified as the main reason for under-reporting [179]. Telemedicine, rapidly expanded to ensure continued access, brought its own safety risks: legal reviews of pandemic-era telehealth highlight frequent deviations from care standards, breaches of privacy, inadequate staff training, and technology failures, all of which can lead to diagnostic and therapeutic errors [171].

Overall, recent studies have shown that COVID-19 not only added to the existing pressure on health systems but also worsened already present issues related to patient safety. Factors such as overload, rapid organizational changes, and clinician burnout increased the likelihood of medication errors, diagnostic delays, and misdiagnoses. In contrast, widespread disruptions in medical care have increased the harm caused by these errors. Some interventions, like structured risk stratification to maintain urgent diagnostics, systematic safety reviews, and digital multidisciplinary meetings, have proven, however, that proactive, system-based strategies are able to decrease error risks, even during a pandemic [180,181].

### 5.1. How Mental Health of the Healthcare Workers Affects Performance

The decline in mental health among clinicians impacts their performance through closely linked cognitive, emotional, and physiological factors. Burnout and post-traumatic stress symptoms weaken the neurocognitive systems essential for maintaining attention, making complex decisions, and monitoring errors accurately. This deterioration increases the likelihood of medical errors over time.

A comprehensive meta-analysis of 13 studies involving 20,643 physicians revealed that overall burnout is linked to nearly a threefold rise in self-reported medical errors (OR = 2.72, 95% CI 2.19–3.37). Emotional exhaustion, depersonalisation, and low personal accomplishment each independently predicted increased error risk [182]. Likewise, a survey of 6586 US physicians found that those who reported a major medical error in the past three months had significantly higher burnout rates (77.6% vs. 51.5%) and fatigue levels (46.6% vs. 31.2%). After adjustments, burnout remained an independent predictor of errors (OR = 2.22, 95% CI 1.79–2.76) [183]. Among American surgeons, each point increase on a depersonalisation scale (0–33) raised the odds of reporting a major error by 11%, and each point increase in emotional exhaustion (0–54) by 5%. This illustrates a graded, rather than threshold, relationship between distress and unsafe performance [184].

These behavioral findings support converging evidence that burnout affects multiple cognitive areas. A multivariate meta-analysis of 17 studies with 730 patients and 649 controls found that clinical burnout relates to small-to-moderate deficits across several domains: episodic memory (g = −0.36), short-term and working memory (g = −0.36), executive function (g = −0.39), attention and processing speed (g = −0.43), and fluency (g = −0.53) [185]. Longitudinal research on stress-related exhaustion shows that impairments in speed, attention, and memory can last at least three years, with patients still performing worse than controls during follow-up [186]. In working populations experiencing non-clinical burnout, objective tests indicate poorer performance on central executive and visuospatial working memory tasks, with dual-task difficulties linked to lower work performance [187]. Even early, non-clinical burnout among healthcare workers has been linked to specific executive attention deficits, such as increased impulsivity during continuous attention tasks [188].

Neurobiological models illustrate how these deficits lead to error-prone practice. Chronic, uncontrollable stress damages prefrontal cortex networks that manage top-down regulation of thought and emotion. This results in a loss of prefrontal gray matter connections under ongoing stress, with some recovery after relief [189]. Such vulnerability of prefrontal circuitry directly impacts abstract reasoning, higher-order decision-making, and the capacity to sustain goal-oriented attention in complex clinical settings.

Emotional dysregulation acts as a parallel pathway from mental health issues to performance failures. Burnout is consistently linked to high rates of depression, anxiety, and sleep problems, all of which impair concentration and decision-making [190,191]. There seems to be a strong association between burnout and depressive (r = 0.74) and anxiety symptoms (r = 0.58). Physicians experiencing burnout may be up to 2.3 times more likely to die by suicide than the general population, highlighting severe affective dysregulation [192]. Depersonalization, a cynical detachment aspect of burnout, predicts higher adverse-event rates and lower perceived care quality among nurses and doctors. Increased depersonalization scores are associated with an increased risk of adverse events (e.g., OR 1.06–1.08 per point in nurses during COVID-19), whereas low personal accomplishment is associated with a modest reduction in medication errors [193,194].

Sleep disturbance provides a third, partially independent, mechanism linking mental health to error. Stress-related exhaustion and burnout are strongly associated with insomnia and non-restorative sleep, which in turn predict later exhaustion and poorer work performance [187,190]. In a survey of 7538 physicians, increasing sleep-related impairment showed a clear dose–response association with clinically significant medical errors: compared with low impairment, moderate, high, and very high levels increased error odds by 53%, 96% and 97%, respectively, even after adjusting for burnout [184]. Experimental and observational data link inadequate sleep with decrements in vigilance, attention, working memory, operative dexterity, and diagnostic accuracy, as well as riskier clinical decision-making [184]. These findings align with broader cognitive-psychology evidence that sleep loss induces dose-dependent attentional lapses and emotional dysregulation via disrupted connectivity between prefrontal regions and limbic structures.

### 5.2. Empirical Evidence Linking Mental Health to Medical Errors

A review of 46 studies found that, across 30 burnout studies and 27 well-being studies, higher burnout or poorer mental health was significantly associated with increased medical errors or worse safety outcomes [195]. Specifically, 83.3% of studies focusing on burnout and 88.9% on wellbeing of healthcare workers showed associations with poorer safety, including increased self-reported errors, adverse events, and near misses [195]. Another meta-analysis of 13 studies involving 20,643 physicians found that overall burnout was associated with nearly a threefold increase in self-reported medical errors (OR 2.72, 95% CI 2.19–3.37). Emotional exhaustion, depersonalization, and low personal accomplishment each independently predicted error risk [182]. Regarding surgeons, a meta-analysis of 27,248 participants found burnout doubled to tripled the risk of medical error involvement (OR 2.51, 95% CI 1.68–3.72). Higher emotional exhaustion was linked to a 71% higher chance of being involved in a patient safety incident (OR 1.71, 95% CI 1.35–2.16) [196]. Additional evidence from a US survey of 6586 physicians across multiple specialties showed those reporting major errors in the past three months had a significantly higher burnout rate (77.6% vs. 51.5%), and burnout remained independently associated with errors after adjustments (OR 2.22, 95% CI 1.79–2.76) [183].

Research done on nurses and about perioperative care showed that these issues extend beyond physicians. A recent meta-analysis of 85 studies with 288,581 nurses revealed that burnout is significantly linked to poorer safety outcomes, including a lower safety climate (SMD −0.68, 95% CI −0.83 to −0.54), reduced safety ratings (SMD −0.53, 95% CI −0.72 to −0.34), and increased nosocomial infections (SMD −0.20, 95% CI −0.36 to −0.04), patient falls (SMD −0.12, 95% CI −0.22 to −0.03), medication errors (SMD −0.30, 95% CI −0.48 to −0.11), adverse events (SMD −0.42, 95% CI −0.76 to −0.07), and missed care (SMD −0.58, 95% CI −0.91 to −0.26) [197]. Evidence from narrative reviews in the operating room shows burnout rates as high as 10–83% among surgeons, anesthesiologists, and OR nurses, with burnout associated with communication issues, operational inefficiencies, and a 2.5 times higher risk of surgical errors in earlier studies [196,198].

Post-traumatic stress symptoms introduce an additional, often compounding, risk of errors. A systematic review by d’Ettorre et al. found that PTSD symptoms are common among hospital staff and are linked to medication mistakes and decreased care standards, although detailed error estimates are less established than those for burnout [164]. In pediatric environments, 72.8% of 445 staff reported at least one incident involving a medical or nursing error, and these events were significant risk factors for later PTSD symptoms; 25.2% met provisional PTSD criteria [199]. Overall, PTSD in healthcare workers is consistently associated with lower quality of care, higher absenteeism, and greater staff turnover, all of which indirectly threaten patient safety [145].

The COVID-19 pandemic served as a natural stress test for these relationships. Narrative and scoping reviews reveal that increased workload, moral distress, and resource shortages during the pandemic led to significant rises in burnout and PTSD. There is also emerging evidence of a concurrent increase in adverse events and safety incidents [141,200,201]. For instance, the prevalence of mild to severe PTSD among ICU staff increased from pre-pandemic levels of 3.3–24% to 16–73.3% during COVID-19. Studies highlight PTSD as a factor contributing to impaired concentration, intrusive thoughts, and lower care standards [145]. Early surveys of emergency physicians indicated probable PTSD rates of about 22%, with high symptom burdens such as sleep disturbances and emotional detachment—factors associated with an increased likelihood of errors [202].

While most empirical research is observational and often depends on self-reported errors, several consistent patterns bolster causal claims. Firstly, meta-analytic effect sizes tend to be moderate to large across various contexts and professions [182,195,196,197]. Secondly, dose–response patterns are frequently observed: for instance, a one-point increase in emotional exhaustion or depersonalization scores correlates with a 5–10% higher likelihood of medical errors [183,203]. Thirdly, a few longitudinal studies indicate a bidirectional cycle where burnout predicts subsequent errors and errors further exacerbate burnout and depressive symptoms [195]. Lastly, using objective safety indicators, such as chart reviews, infection rates, or medication errors, has shown that associations with mental health, especially depression, stress, and overall burnout, remain evident, though sometimes weaker [195,197].

Mental health issues and medical errors are intertwined in a self-perpetuating cycle, where distress both leads to mistakes and is intensified by them. When clinicians are involved in malpractice claims, they often experience the ‘second victim’ phenomenon, characterized by intense emotional, cognitive, and physical reactions similar to post-traumatic stress disorder, which can trigger or worsen burnout [8,204,205]. Reviews estimate that 9% to 50% of healthcare professionals will experience second-victim symptoms at least once, with point prevalence ranging from 10.4% to 43.3% across samples [121,206,207]. In a North-African survey, 68.2% of physicians reported making a medical error, and 23.5% met criteria for PTSD on the Impact of Event Scale–Revised (cut-off >33), with higher risk among women and those involved in serious events [208].

Second victim experiences share a symptom profile closely aligned with core PTSD symptoms. An integrative review of 19 studies found that 60% of impacted professionals reported negative changes in cognition and mood, particularly guilt, shame, and self-doubt. Additionally, 35% experienced anxiety, 30% insomnia, and 20% intrusive memories, patterns that closely resemble DSM-5 PTSD criteria [12]. A systematic analysis performed by Seys et al. has revealed that involvement in adverse events has consistently triggered feelings of embarrassment, guilt, anxiety, self-doubt, and hypervigilance, along with physical symptoms such as tachycardia, fatigue, and sleep disturbance [8,209]. A recent narrative review by Gibalska et al. also highlighted persistent guilt, depressive symptoms, suicidal thoughts, sleep and eating disorders, and professional burnout after errors, with signs of potential progression to full PTSD [207]. These reactions undermine their confidence in themselves, erode trust in their clinical abilities, and cause avoidance of similar situations, eventually impacting their well-being and professional performance.

Research shows that error-related trauma is not just a result of burnout, but also a factor that contributes to ongoing distress, creating a harmful cycle. A 2019 emergency medicine review described this as a “continuous chain of events”: burned-out physicians are more prone to errors, which then lead to second victim syndrome, legal stress, and more burnout, increasing risks of depression, substance abuse, leaving the profession, or suicide [210]. A study of 393 North-African doctors found that experiences as a second victim were closely linked to career stress, anxiety, burnout, and fear of new mistakes, illustrating how psychological effects after an error can raise the chance of future mistakes and defensive medical practices [208]. Another review indicated that second victim syndrome hampers work performance and quality of care, promotes defensive medicine, increases costs, and may heighten the risk of additional adverse events [207].

### 5.3. Confounding Factors in the Mental Health—Medical Error Relationship

Confounding factors substantially obstruct the ability to directly associate clinicians’ mental health problems with medical errors. Healthcare systems are intricate, with organizational stressors and personal vulnerabilities intertwining, making it difficult to isolate the effects of a clinician’s psychological state from environmental factors.

System-level issues, including understaffing, time pressure, limited resources, or poorly designed workflows, are known to be closely associated with decreased/altered mental health in medical personnel and also with decreased patient safety. During COVID-19, large surveys and reviews have highlighted how surge conditions have intensified these pressures: up to 47% of healthcare workers have experienced burnout, 38–42% suffered from anxiety, 33–34% faced depression, and approximately 30–32% experienced acute stress or post-traumatic symptoms [3,211,212]. Many also worked long hours, with inadequate PPE and rapidly evolving protocols [3,211,212]. Early-pandemic cross-sectional data from nearly 1800 staff members showed that caring for COVID-19 patients nearly doubled the odds of burnout (adjusted OR 1.87), and working in acute or critical care increased burnout risk by about 30% [213]. These same system stressors are independently associated with higher error rates and lower patient safety, indicating that the link between mental health and errors is heavily influenced by organizational factors [141,214] (Restauri and Sheridan, 2020; Fatani et al., 2024).

The COVID-19 pandemic clearly exemplifies this interconnectedness. Frontline healthcare workers experienced extreme workloads, role changes, supply chain issues, and breakdowns in communication. In one multinational ICU study, over 40% of staff reported increased workloads, inadequate social support, and poor communication, with burnout rates over 50% and 27% showing probable PTSD [160]. Other pandemic-era studies using structural equation modelling indicate that deficiencies in preparedness, communication, and supply chains directly compromised care quality. Staff mental health was found to be one of the most significant factors influencing perceived disruptions [214]. Since system failures both worsen working conditions and contribute to anxiety, depression, and burnout, it remains complex to differentiate errors caused by mental health issues from those due to unsafe systems or their interaction.

Individual resilience and psychosocial resources add complexity to causal inference. Evidence from meta-analyses and quick reviews shows that resilience, social support, effective coping mechanisms, and psychosocial resources help buffer the mental health impacts of pandemic-related stressors and decrease burnout. Conversely, high infection risk, long working hours, and direct COVID-19 exposure tend to increase these problems [212,215]. In large samples, resilience partially mediates the link between depression and burnout, weakening this connection, while social and organizational support can protect against all three burnout domains [212]. Interestingly, many clinicians experiencing high burnout or post-traumatic symptoms continue to perform competently, whereas others show significant functional decline even at lower distress levels [65,216]. Traits like agreeableness, mindfulness, and psychological well-being are negatively linked to burnout, whereas time pressure and workload are positively linked, leading to considerable variability in how mental health issues affect performance and errors [217].

Measurement issues may also significantly contribute to confounding. Most research depends on self-reported mental health symptoms and medical errors, both prone to recall bias, selection bias, and social desirability. The literature is mainly composed of cross-sectional internet surveys, which limit the ability to infer causality and obscure the temporal relationship between distress and errors [212,218]. Clinicians experiencing burnout, anxiety, or depression may be more self-critical, thus more likely to identify and report errors; conversely, shame, fear of blame, and organizational cultures emphasizing perfectionism can suppress disclosures, especially among highly stressed staff [29,219]. For instance, one US study found 95% of residents reported at least one self-perceived error, but only 32% engaged in formal debriefings, with fear of retaliation and shame acting as key barriers to open discussion [219]. During pandemic surges, routine reporting systems and formal safety procedures were often disrupted, further diminishing the reliability and comparability of error data [1,220].

Despite these methodological challenges, evidence consistently shows a genuine yet complex causal link between worsening mental health and a higher risk of medical errors. A recent meta-analysis found that physician burnout is significantly associated with increased self-reported medical errors, as well as higher rates of turnover, substance abuse, and suicidality [141]. In a national survey of Chilean doctors during COVID-19, burnout scores independently predicted more days of medical leave, intentions to leave, and perceived errors, even after controlling for demographic, personality, and work environment factors [217]. Cross-sectional surveys and reviews regularly connect depression, anxiety, acute stress, and PTSD with impaired concentration, lower job performance, and compromised patient safety. Large-scale surveys also show that around 37–47% of healthcare workers experienced burnout, with about one-third suffering from depression and anxiety during the pandemic—levels much higher than in the general population [3,211,218].

Longitudinal and mechanistic data are limited, and most existing studies lack independent, objective error measurements or rigorous control of confounding factors such as staffing, workload, and case diversity [212]. Nonetheless, evidence from various research methods, such as large observational studies, meta-analyses, rapid reviews, structural modeling, and qualitative accounts of error-related stress, indicates that mental health issues are more than just associated; they actively increase the risk of medical errors, especially during periods of extreme system stress like the COVID-19 pandemic. From a policy and patient safety perspective, this indicates that enhancing staffing levels, managing workload, fostering a positive organizational culture, and improving error-reporting systems are all closely connected to protecting clinicians’ mental health. These improvements are crucial for reducing medical errors and sustaining those reductions.

Some practical recommendations for addressing healthcare worker burnout and PTSD leading to medical errors are presented in Table 4.

## 6. Legal Implications

The threat and reality of malpractice litigation amplify this vicious cycle. Malpractice claims are frequent: by age 65, up to 75% of physicians in low-risk specialties and 99% in high-risk specialties will have faced at least one claim [139]. Litigation acts as a powerful chronic stressor, often called “medical malpractice stress syndrome,” and is marked by anxiety, depression, insomnia, and defensive practice changes [16,230,231]. In a survey of 282 physicians with recent claims, 56.4% experienced significant psychological reactions, and 45.4% reported practice modifications, such as perceiving patients as potential plaintiffs (45.4%), ordering more tests (36.2%), or avoiding certain patients (21.6%) or procedures (19.9%). Those experiencing psychological effects showed more symptoms and were more likely to adopt defensive behaviors [232]. Among 7164 American surgeons, 24.6% had been involved in a malpractice suit within the past two years; this was strongly linked to burnout, depression, and recent suicidal thoughts (all *p* < 0.0001). In multivariable analyses, both depression (OR 1.27, *p* = 0.0003) and burnout (OR 1.17, *p* = 0.0306) remained independently associated with recent malpractice involvement. Affected surgeons reported lower career satisfaction and were less likely to recommend medicine as a career [233].

These findings are consistent across various specialties and settings. A survey of 353 Turkish orthopaedic and trauma surgeons found that 65.4% were currently facing at least one malpractice claim. Those under investigation reported significantly higher emotional exhaustion, greater hesitancy in clinical behavior, and notably lower job satisfaction [234]. In Taiwan, a study of 1206 primary care physicians found that 25.2% had experienced a malpractice dispute. After propensity score matching, those involved in disputes showed significantly poorer overall health (mean difference −4.85, 95% CI −7.61 to −1.80), mental health (−2.68, 95% CI −5.03 to −0.34), and vitality (−3.28, 95% CI −6.10 to −0.47) on SF-36 measures [235]. Narrative reviews from different regions highlight similar patterns: legal cases lead to stages of shock, denial, anger, depression, and eventual acceptance, with documented instances of suicide following negligence accusations [31]. A large Chinese cross-sectional study involving 1031 doctors and nurses found that high fear of malpractice nearly tripled burnout odds (OR 2.87, 95% CI 1.94–4.23), suggesting that even anticipatory legal anxiety can significantly harm mental health and increase error risk indirectly [236].

Without organizational support, clinicians might adopt unhealthy coping mechanisms, such as withdrawal, avoiding high-risk patients, or overusing defensive testing, which compromise safety, increase costs, and sustain the cycle [8,121]. However, growing evidence shows that structured peer support and second victim programs can lessen immediate distress and feelings of isolation. This suggests that system-level strategies to interrupt this cycle are vital to clinician well-being and patient safety policies [206,237].

Healthcare institutions increasingly face legal accountability for systemic contributors to medical error, beyond the liability of individual clinicians. The doctrine of corporate negligence, articulated in landmark U.S. cases such as Darling v. Charleston Community Memorial Hospital and expanded in later decisions, recognizes hospitals’ direct, non-delegable duties to provide safe facilities, appropriately staff units, rigorously credential and privilege clinicians, and monitor the quality of care delivered within their walls [238,239]. Under this doctrine, hospitals may be held liable for failures such as chronically unsafe nurse-to-patient ratios that predictably increase mortality and errors, unsafe productivity pressures that drive clinicians beyond reasonable workloads, and negligent hiring or retention when institutions overlook patterns of incompetence, impairment, or burnout [240]. Vicarious liability under the doctrine of respondeat superior further extends institutional responsibility to the negligent acts of employees occurring within the scope of employment, while the emerging concept of apparent agency may impose liability even for independent contractors whom patients reasonably perceive as hospital staff [241,242]. Contemporary medico-legal analysis further emphasizes that many adverse events arise from organizational failures, including understaffing, inadequate supervision, defective policies. These are often more important, at least in some circumstances, than isolated individual incompetence, underscoring a legal and ethical imperative for health systems to address modifiable workplace and safety conditions [243,244].

Addressing the intersection of clinician mental health and medical liability requires comprehensive policy reform at institutional, regulatory, and legislative levels. The 2022 U.S. Surgeon General’s Advisory on Health Worker Burnout called for systemic changes including elimination of punitive policies for seeking mental health care, protection from workplace violence, adequate staffing mandates, and investment in support programs [245]. Several evidence-based reform approaches merit consideration: communication and resolution programs, which encourage transparent disclosure, early compensation for injured patients, and system-level learning without adversarial litigation, which were shown to decrease both claim frequency and defense costs [246]. No-fault administrative compensation systems, which were successfully implemented in country such as New Zealand, Sweden, and Denmark, separate patient compensation from fault determination, thereby reducing defensive medicine while improving access to redress for injured patients [27,247]. Safe harbor provisions protecting clinicians who follow evidence-based guidelines, reform of licensing board mental health disclosure requirements that currently deter help-seeking, and mandatory organizational wellness metrics in accreditation standards represent additional policy levers [248].

## 7. Limitations

This article has a series of relevant limitations, which include: (1) Narrative review design—as a narrative rather than systematic review, this synthesis did not employ formal meta-analytic techniques or comprehensive quality assessment tools such as GRADE or Newcastle-Ottawa Scale. This may introduce selection bias in study inclusion. (2) Language restriction—only English-language publications were included, potentially excluding relevant research from non-English speaking regions, particularly given the global nature of the pandemic. (3) Search strategy—while multiple databases and supplementary methods (reference screening, AI-assisted literature discovery) were employed, the search was primarily PubMed-based, potentially missing studies indexed only in other databases (e.g., PsycINFO, CINAHL, Embase). (4) Limitations of the included literature—most studies examining burnout, PTSD, and medical errors are cross-sectional, limiting causal inference. Few longitudinal studies track the temporal relationship between mental health deterioration and subsequent errors. The cross-sectional design of most included studies means that observed associations could reflect reverse causation (e.g., clinicians experiencing distress may be more likely to perceive and report errors) or shared confounders. The causal pathways presented should be interpreted as working hypotheses supported by converging evidence rather than definitively established mechanisms. (5) Self-reported outcomes—most studies rely on self-reported burnout, PTSD symptoms, and medical errors, introducing recall bias and social desirability effects. Objective error measurements (e.g., chart reviews, incident reports) are underrepresented. Importantly, studies using self-reported errors consistently show stronger associations with burnout than those using objective measures, suggesting potential measurement artifact (6) heterogeneity in definitions and instruments—burnout measurement varies widely (MBI variants, CBI, single-item measures), and PTSD is assessed using different thresholds (IES-R, PCL-5, clinical diagnosis), limiting comparability across studies. (7) Geographic concentration—studies are disproportionately from high-income countries (USA, Europe, China), with lower representation from low- and middle-income settings where healthcare system strain may differ substantially. (8) Pandemic-specific confounders isolating the effects of mental health from pandemic-specific system disruptions (supply shortages, protocol changes, staffing crises) is challenging, as both contribute to error risk. (9) Publication bias: studies reporting significant associations between burnout/PTSD and errors may be more likely to be published than null findings. (10) Temporal limitations—the pandemic’s mental health effects continue to evolve, and post-pandemic recovery trajectories are only beginning to be documented. Long-term follow-up data remain limited. (11) The medico-legal and patient safety implications rely heavily on the robustness of the association between clinician psychological distress and error occurrence. However, a substantial proportion of studies use self-reported errors or perceived safety incidents rather than objective clinical outcomes.

## 8. Conclusions

The COVID-19 pandemic has augmented a continuous cycle in which burnout leads to more medical errors, which then may precipitate adverse events, second victim reactions, and malpractice disputes. These, in turn, is associated with PTSD symptoms that further impair cognition and worsen burnout. This cycle is evidenced by nearly a threefold rise in errors among burned-out doctors and the high overlap between burnout and PTSD (86–98%) in specific high-risk cohorts during pandemic surges; these extreme figures should not be generalized to all healthcare worker populations. It cannot be broken solely through individual resilience training. System-level solutions such as peer support, structured second victim teams, blame-free error reporting, and workload management are essential to safeguard clinician wellbeing and patient safety. The evidence base has robustly established the high prevalence of burnout and PTSD among healthcare workers, the association between burnout and self-reported medical errors (OR ~2.5–3.0), key risk factors including excessive workload and inadequate resources, and the bidirectional relationship between burnout and PTSD.

However, critical gaps persist. Causal mechanisms linking clinician distress to patient harm remain incompletely understood, requiring longitudinal studies with objective performance measures rather than cross-sectional self-report designs. Fewer than 10% of burnout interventions have been evaluated through randomised controlled trials, and economic analyses demonstrating return on investment are largely absent. Evidence is geographically concentrated in high-income Western settings, limiting generalisability to resource-constrained environments. Additionally, the relationship between clinician mental health and actual malpractice outcomes remains poorly documented. 

Promising directions include wearable technology for real-time stress monitoring, machine learning models for burnout prediction, biomarker studies linking chronic stress to cognitive performance, and implementation science approaches to scaling effective interventions across diverse healthcare systems.

## Figures and Tables

**Table 1:** Research methodology for the initial search.

Topic	Methodology	PubMed Keywords
1. Burnout in Healthcare Professionals	Systematic review and meta-analysis of burnout prevalence, dimensions (emotional exhaustion, depersonalization, reduced personal accomplishment), and consequences across healthcare settings	“burnout” AND “healthcare workers” “Maslach Burnout Inventory” AND (“physicians” OR “nurses”) “emotional exhaustion” AND “medical professionals” “burnout” AND “COVID-19” AND “healthcare” “depersonalization” AND “clinical practice” “professional efficacy” AND “burnout syndrome” “burnout” AND “patient safety”
2. Burnout Characteristics During the COVID-19 Pandemic	Comparative analysis of pre-pandemic versus pandemic burnout rates; examination of moral injury and ethical distress as pandemic-specific phenomena	“burnout” AND “COVID-19 pandemic” AND “healthcare” “moral distress” AND “pandemic” AND “nurses” “frontline workers” AND “burnout” AND “SARS-CoV-2” “redeployment” AND “burnout” AND “healthcare workers” “moral injury” AND “COVID-19” AND “physicians” “burnout” AND “intensive care” AND “pandemic” “ethical distress” AND “healthcare” AND “coronavirus”
3. Post-traumatic Stress Disorder in Healthcare Workers	Systematic review of PTSD prevalence, diagnostic criteria (DSM-5, ICD-11), trauma exposure pathways, and differentiation from acute stress disorder, vicarious trauma, and secondary traumatic stress	“PTSD” AND “healthcare workers” “post-traumatic stress disorder” AND (“nurses” OR “physicians”) “secondary traumatic stress” AND “medical staff” “vicarious trauma” AND “healthcare professionals” “workplace violence” AND “PTSD” AND “hospital staff” “critical incidents” AND “trauma” AND “emergency medicine” “trauma exposure” AND “first responders” “acute stress disorder” AND “healthcare”
4. The Overlap Between PTSD and Burnout	Cross-sectional and longitudinal analysis of comorbidity patterns, shared risk factors, and bidirectional relationships between PTSD and burnout	“PTSD” AND “burnout” AND “healthcare” “comorbidity” AND “trauma” AND “occupational stress” “second victim” AND “burnout” AND “PTSD” “bidirectional relationship” AND “mental health” AND “clinicians” (“PTSD” OR “post-traumatic stress”) AND “emotional exhaustion” “trauma” AND “depersonalization” AND “healthcare workers”
5. Impact of COVID-19 on PTSD in Healthcare Workers	Meta-analysis of pandemic-related PTSD prevalence, comparison with pre-pandemic rates, and analysis of COVID-specific stressors (PPE shortages, infection fear, mass death exposure)	“PTSD” AND “COVID-19” AND “healthcare workers” “pandemic” AND “post-traumatic stress” AND (“ICU staff” OR “intensive care”) “frontline workers” AND “psychological trauma” AND “coronavirus” “PPE shortage” AND “mental health” AND “pandemic” “infection fear” AND “PTSD” AND “healthcare” “mass casualty” AND “trauma” AND “COVID-19” “pandemic stress” AND “nurses” AND “PTSD”
6. The Link Between Mental Health and Medical Error	Systematic review examining causal pathways, mechanisms (cognitive, emotional, physiological), and types of errors associated with mental health conditions	“medical errors” AND “burnout” AND “physicians” “patient safety” AND “mental health” AND “healthcare workers” “diagnostic errors” AND (“cognitive impairment” OR “stress”) “adverse events” AND “burnout” AND “nurses” “medication errors” AND “fatigue” AND “healthcare” “medical mistakes” AND “psychological distress” “preventable harm” AND “clinician wellbeing”
7. How Mental Health of Healthcare Workers Affects Performance	Analysis of cognitive neuroscience evidence, neurobiological pathways, and performance metrics linking mental health decline to impaired clinical decision-making and error monitoring	“cognitive function” AND “burnout” AND “healthcare” “decision-making” AND “stress” AND “physicians” “executive function” AND “exhaustion” AND “medical professionals” “sleep deprivation” AND “medical errors” “attention” AND “burnout” AND “clinical performance” “working memory” AND “occupational stress” AND “healthcare” “prefrontal cortex” AND “chronic stress” AND “cognition” “emotional dysregulation” AND “burnout”
8. Empirical Evidence Linking Mental Health to Medical Errors	Meta-analysis and systematic review of observational studies quantifying associations; analysis of second victim phenomenon and malpractice-related psychological distress	“self-reported errors” AND “burnout” AND “meta-analysis” “patient safety incidents” AND “PTSD” AND “healthcare” “second victim phenomenon” AND “medical errors” “malpractice” AND “psychological distress” AND “physicians” (“adverse events” OR “safety incidents”) AND “mental health” AND “nurses” “surgical errors” AND “burnout” AND “surgeons” “medication errors” AND “emotional exhaustion” “second victim” AND “trauma” AND “healthcare”
9. Confounding Factors in the Mental Health—Medical Error Relationship	Critical analysis of system-level factors (staffing, workload, resources), organizational culture, individual resilience, and methodological limitations (measurement bias, self-report)	“healthcare systems” AND “burnout” AND “COVID-19” “workload” AND “staffing” AND “medical errors” “organizational support” AND “mental health” AND “healthcare” “resilience” AND “burnout” AND “protective factors” “measurement bias” AND “self-reported” AND “burnout” “work environment” AND “patient safety” “staffing levels” AND “adverse events” “organizational culture” AND “safety climate” “confounding factors” AND “burnout” AND “errors”

**Table 2:** Pooled burnout prevalence among healthcare workers and subgroups during COVID-19. HCW—healthcare workers, EE—emotional exhaustion, PA—personal accomplishment.

Population/Review Focus	Sample Size	Burnout Metric and Prevalence	Key Details/Subdomains
All healthcare workers, global [61]	250 studies, *n* ≈ 293,000	Burnout 43.6% (95% CI: 36.3–51.2)	Same meta-analysis: PTSD symptoms 30.6% (23.6–38.5)
All HCWs during COVID-19 (MBI only) [62]	7 studies, *n* = 5022	High/moderate EE: 45% + 37%; DP: 49% + 18%; low PA: 38% + 51%	Severe levels common in all 3 domains
All HCWs, mixed tools [18]	Mixed	Overall burnout 52% (40–63%)	EE 51% (42–61%), DP 52% (39–65%), low PA 28% (25–31%)
Nurses during COVID-19 (updated meta-analysis) [63]	19 studies	Any burnout 59.5%	EE 36.1%; DP 32.4%; low PA 33.3%
Nurses during COVID-19 (MBI-focused, early pandemic) [64]	16 studies, *n* = 18,935	EE 34.1%; DP 12.6%; low PA 15.2%	High heterogeneity
Nurses across health emergencies incl. COVID [65]	176 studies	Overall burnout 48% (42–55%)	Higher in ICU/ED (SMD 0.10) and with COVID exposure
Physicians during COVID-19 [17]	45 studies	Overall burnout 54.6% (46.7–62.2)	Early pandemic 60.7% vs. late 49.3%; frontline OR 1.64 vs. 2nd line
Physicians during COVID-19 [66]	30 studies	Burnout range 6–99.8%	No pooled %; high heterogeneity; highlights tool variability
ICU physicians (pre- and during COVID-19) [67]	18 studies	High-level burnout 41% (0.33–0.50)	EE 28%; DP 33%; low PA 38%
ICU nurses (pre- and during COVID-19) [67]	20 studies	High-level burnout 44% (0.34–0.55)	During COVID: 61% vs. 37% pre-COVID (*p* = 0.003)
ICU and ED HCWs during COVID-19 [68]	11 studies	Overall burnout 49–58%	Nurses at higher risk; shortages, worry, stigma key drivers
Emergency department HCWs (global) [69]	29 studies, *n* = 16,619	Overall burnout 43%; high-risk burnout 35%	High EE 39%, high DP 43%, low PA 36%; higher during COVID
Central Asia HCWs (3 countries) [57]	*n* = 2685	Occupational burnout 28.3%	Severe COVID history OR up to 2.27 for burnout
Polish HCWs, 5 groups [60]	*n* = 2196	Burnout 27.7–36.5% by role; 36.5% in nurses	High stress OR 3.88; traumatic events OR 1.91; mobbing OR 1.83
Oncology HCWs Wuhan (frontline vs. usual wards) [70]	Mixed	Burnout 13% frontline vs. 39% usual wards	FL staff less worried about infection, more team coherence

**Table 3:** Pre-pandemic v. pandemic PTSD Prevalence. Key data.

Population	Pre-Pandemic Prevalence	During COVID-19 Pandemic	Change
Overall Healthcare Workers			
HCWs (general)	15–20%	25–34% (pooled meta-analysis)	+10–14 percentage points [149]
HCWs (severe PTSD)	~10%	14%	+4 percentage points [149]
By Professional Role			
Nurses (general)	14–18%	25–29.1%	Nearly doubled [132,152]
ICU nurses	18–24%	29–51.5%	+11–27 percentage points [144,153]
Physicians (general)	10–15%	18–25%	+8–10 percentage points
Frontline HCWs	15–20%	34–55.2%	Nearly tripled [154]
ICU Staff Specifically			
ICU professionals (mild-severe)	3.3–24%	16–73.3%	Up to 7-fold increase [145]

**Table 4:** Practical recommendations for addressing HcW Burnout and PTSD causing medical errors [56,221,222,223,224,225,226,227,228,229].

Domain	Recommendation
Organizational/Institutional	
Workload Management	Implement maximum patient-to-nurse ratios (recommended 1:4 for medical-surgical units)
	Mandatory minimum 11 h rest periods between shifts
	Limit consecutive shift length to maximum 12 h
	Protected non-clinical time for documentation
Psychological Support Systems	Establish structured peer support programs (e.g., Schwartz Rounds, RISE programme)
	Implement second victim response teams with 24/7 availability
	Provide confidential counselling services with guaranteed anonymity from licensing boards
	Implement regular mental health screening using opt-out model
	Establish critical incident stress debriefing protocols (within 72 h of adverse events)
Culture Change	Implement blame-free error reporting systems (Safety-II approach)
	Mandatory leadership training on psychological safety
	Establish regular communication forums between administration and clinical staff
	Recognise and celebrate near-miss reporting as safety improvement
Individual-Level	
Stress Reduction	Mindfulness-based stress reduction programmes (8-week MBSR)
	Facilitate access to cognitive-behavioural therapy
	Resilience training programmes (with caution against victim-blaming)
Self-Care	Sleep hygiene education and fatigue management
	Peer support group participation
Policy/Regulatory	
Licensing Reform	Reform medical licensing questions regarding mental health treatment history
Legal Protections	Implement safe harbour provisions for good-faith error reporting and disclosure
Compensation Reform	Consider no-fault administrative compensation systems for medical injuries
Accreditation	Mandate organisational wellness metrics in hospital accreditation standards
Research Funding	Dedicated funding for intervention effectiveness research
Research Priorities	
Longitudinal Studies	Track burnout → medical errors → second victim trajectories over time
Intervention Trials	Conduct randomised controlled trials of organisational interventions
Economic Analyses	Perform cost-effectiveness and return-on-investment analyses of wellness programmes
Measurement Validation	Validate burnout and PTSD screening tools across diverse healthcare populations

## Data Availability

The original contributions presented in this study are included in the article. Further inquiries can be directed to the corresponding author.
